# Supplementary material for: Are carbon emissions trading and green financial instruments synergistic? -Comprehensive quantitative research based on content analysis
Source: PLoS One. 2024 Mar 7;19(3):e0298601. doi: 10.1371/journal.pone.0298601 (PMC10919601; doi:10.1371/journal.pone.0298601)
Supplement: S1 Data — (ZIP) [file pone.0298601.s001.zip › Data/地级市Z_F工作报告.zip!/║■▒▒/╢⌡╓▌╩╨/└┤╫╘╣1⁄2╓┌║┼í╛┬φ┐╦╩2╛▌═°í┐_2015.docx]

各位代表：

　　现在，我代表市人民政府向大会作工作报告，请予审议，并请各位市政协委员和列席人员提出意见。

　　一、2014年工作回顾

　　刚刚过去的2014年，是很不平凡的一年。全市上下在省委、省政府和市委的领导下，面对持续加大的经济下行压力，按照“竞进提质、升级增效”的总要求，咬定发展不放松，主动适应经济新常态，统筹推进稳增长、促改革、调结构、惠民生各项工作，取得了好于预期的成绩。全年实现地区生产总值690亿元，同比增长9.5%，增幅居全省第二位；人均生产总值历史性突破1万美元大关；规模以上工业增加值373.15亿元，增长11.3%；全社会固定资产投资698.03亿元，增长22.1%；地方公共财政预算收入42.74亿元，增长11.2%；社会消费品零售总额230.28亿元，增长13.1%；外贸出口1.97亿美元，增长11.8%；实际利用外资2.19亿美元，增长30.2%；城镇居民人均可支配收入22970元，增长10%；农村居民人均纯收入11332元，增长11%。主要指标增幅位居全省前列，其中工业增加值、工业用电量、高新技术产业增加值和农业增加值四项指标增幅均居全省第一位。

　　——工业经济逆势奋进，速度质量同步提升。面对复杂严峻的经济形势，我们不等不靠，出台了促进经济发展、全面深化改革的一系列政策措施，扎实推进重点产业倍增计划，筹措了8.7亿元财政间歇资金，加大银企对接力度和频次，着力解决企业用工、用地等困难，强化生产要素保障，推动工业经济总量扩张、结构改善、质量提升。全市规模以上工业实现销售收入1290亿元，增长9.2%，新增规模工业企业50家，新增销售收入过亿元企业34家。实施技改项目109个，完成技改投资64.5亿元，增长28.1%。全市新增高新技术企业16家，实现高新技术产业增加值100.12亿元，增长16.9%，占GDP比重达到14.5%。生物医药、电子信息等新兴产业比重同比提高2.16个百分点。连续第6年被省政府评为质量先进市。

　　——生态农业亮点纷呈，农村发展势头喜人。编制完成全市生态农业发展规划，引领生态农业迅猛发展。推动农民以“带地入股、保底分红”模式进行土地流转，着力培育新型农业经营主体，新增家庭农场210家，新增农村专业合作组织54家，70%以上的农户进入合作服务体系，全市面积100亩以上的生态农业示范基地达到129个。“梁心”有机番茄、“梁道”有机稻米等一批具有鄂州特色的生态农业品牌兴起，新增有机农产品认证17个、农产品地理标志证明商标6个、国家级现代水产种业示范场1个和省部级水产健康养殖示范场8个。我市获批创建全省首家生态农业标准化示范区，台湾生态农业创业园、省农科院科技创新基地落户我市。粮食和水产品产量分别达到36.38万吨、45万吨，双双实现“十一连增”。农产品加工业再上台阶。实现农产品加工值280亿元，增长33%。城乡一体化发展加快。大力开展美丽乡村建设。编制完成32个美丽乡村规划，涂家垴镇万秀村等6个村获评全省“绿色示范乡村”。加大农田水利、安全饮水、国土整治等农业基础设施建设投入力度，农村生产生活条件进一步改善。

　　——第三产业成长加快，新兴业态迅速崛起。把发展现代服务业作为调结构的主攻方向，以电子商务、智慧物流为代表的现代服务业为经济发展注入新活力。全市限上企业网络零售额占全省限上企业网络零售额的23%。葛店开发区电子商务基地快速成长，引进了苏宁易购、上海易商等大型电商企业，唯品会当年动工建设并投入运营，当年上交税收1.2亿元，实现网络零售额30.5亿元。现代物流、信息服务等现代服务业呈现竞相发展的良好态势，东方甩挂物流联盟、宝湾国际、潮团国际等知名企业落户我市。推动金融创新，支持实体经济发展。全市金融机构各项贷款余额303亿元，增长11.8%。鄂州农村信用合作社顺利完成改制。我市连续第10年荣获全省“金融信用市”称号。文化旅游、生态旅游业发展加快，全年接待国内外游客575万人次，实现旅游总收入50.3亿元，分别增长19.02%、21.09%。

　　——各项改革稳步推进，创新活力日益显现。全面推进重点领域和关键环节改革。简政放权，深化行政审批制度改革，审批事项精简至92项，审减率达73%，18类39个审批项目实行零收费。在新区成立行政审批局和综合执法局，享有市级审批和执法管理权限。在全省率先实行工商注册资本认缴制和“先照后证”登记制度改革，实施“一址多照”登记制度。通过改革催生市场主体蓬勃发展，全年新登记市场主体8990户，其中私营企业2011户，增长19.14%；新增企业注册资本57亿元，增长100.05%。稳步推进财税体制改革，严格预算管理，推进公务卡结算制度，部门公用经费下降15%；归集整合各单位结余资金，建立了生态农业、高新产业、现代服务业三项产业基金，助推相关产业发展。企业在资本市场直接融资取得突破，13家企业在“新三板”和武汉股权托管交易中心挂牌。加快国有资产管理运营制度改革，对行政事业单位资产清理归集，实行市场化运营。深入推进农村产权制度改革，完成16个乡镇、216个村土地确权登记颁证工作。扩大农村“五权”抵押融资，共发放贷款8254万元。规范推进城乡建设用地挂钩指标交易，成交额2123万元。持续推动公共服务下沉，服务范围扩大到84个城乡社区。事业单位分类改革、公立医院改革、家庭经济状况核对试点、社会组织直接登记等改革也取得新进展。

　　——新区开发全面提速，发展后劲更加充足。我们抢抓国家推进长江经济带开放开发重大历史机遇，加快推进沿江滨湖新区开放开发。出台扶持新区发展的13条政策，赋予新区市级行政管理权限。加快建设大平台，谋划大项目，培植大产业，打牢发展基础。各新区基础设施建设全面提速，八大新区完成基础设施建设投资94亿元，实际开发面积突破100平方公里，葛店开发区、鄂州开发区、花湖开发区、梧桐湖新区、红莲湖新区框架格局基本形成。各新区围绕主导产业开展产业招商，新落地亿元项目85个，其中5亿元以上项目15个。总投资74亿元的鄂州电厂三期、20亿元的南山集团临港新城、16亿元的南都新能源和投资均超过10亿元的依讯北斗、物联网启动建设；东湖高新创意科技城、彤翰电子、华中数控、建华管桩等57个亿元项目建成投产。

　　——建设管理水平提升，城市面貌明显改观。着力完善城市功能，提升城市品位。创新城市规划编制与管理，我市被列为全国28个“多规合一”试点城市。加大城市配套设施投入，主城区实施市政基础设施建设项目49项，完成投资15亿元，投资额创历史新高。吴楚大道西段、葛山大道改造等项目进展顺利，鄂州大道南互通、三江港铁路货运站主体工程竣工，火车站广场投入使用，城际铁路开通运营。桔园生态公园、寒溪生态广场即将开园，葛华水厂竣工供水。扎实开展“五城同创”，集中开展城市管理专项整治和爱国卫生大扫除等专项行动，在主城区实施“禁鞭”。改造升级15个城区集贸市场，对明堂市场、古楼街等地段集中开展了重点整治，城区卫生状况明显改善。完成主城区物业管理全覆盖三年目标任务。统筹推进各专项创建工作，顺利通过省级文明城市检查验收、国家园林城市复查考评、省级环保模范城中期评估、城区除“四害”省级考核评估，第六次蝉联省级文明城市，正式入围第三批创建全国旅游标准化试点城市。

　　——生态建设成效显著，人居环境不断优化。大力整治工业污染，实施球团厂烧结烟气脱硫、电厂1#2#机组脱硝、世纪新峰脱硝等重点工程，关停德胜钢铁等小水泥、小粉磨、小铸造企业74家，淘汰落后炼铁产能76万吨、水泥产能60万吨。强化农业点源污染防治，完成31家规模养殖场污染治理工程。开展环保部综合督查试点工作，建立了环保监管长效机制。梁子湖区全面退出一般工业，实施涂镇湖破堤还湖、洋澜湖综合治理、主城区雨污分流等工程。

　　推进全国水生态文明示范市建设，对全市湖泊逐一编号造册，实行“湖长制”管理。江湖连通、湖港连通工程顺利推进。推进环梁子湖生态景观林带、“三边”植树建设，新增造林面积7万亩。加快推进城乡污水全收集、全处理，红莲湖污水处理厂即将投入运行，我市被列为全省城乡污水一体化处理试点城市。

　　——民生保障明显改善，社会事业全面进步。坚持民生决定目的，持续加大民生领域投入，民生类支出占全市财政总支出70%以上。筹资3.6亿元，完成“十件实事”。坚持以创业带动就业，全市城镇新增就业2.6万人，城镇登记失业率3.8%，低于目标控制数。企业退休人员连续十一年提高养老金。五项基本社会保险新增扩面3.2万人次，实现了医保即时结算和异地结算。在全省率先实现被征地农民就业和社会保障全覆盖，全市1.86万名60周岁以上被征地农民每月领取240元养老金。在全省率先对城乡“五类”特困群体实行兜底保障，惠及困难群众2.2万人次。完成7个城市社区居家养老和127个农村互助养老示范点建设，改造升级10个农村福利院。完成320个村民委员会选举换届。巩固义务教育均衡发展成果，筹资8737万元，实施义务教育学校标准化建设和薄弱学校改造工程，华容区、梁子湖区义务教育均衡发展顺利通过国家检查验收；实施教育信息化工程，被确定为全国教育装备实验区和全省教育信息化试点市。大力改善医疗卫生条件，市中心医院、精神卫生中心等改扩建工程进展顺利；武汉大学人民医院托管市中心医院，让全市人民享受更高水平的医疗服务。启动实施“单独两孩”政策，出生人口性别比稳步下降。大力推进周周乐、周周演、送戏下乡、农家书屋、村村响、农村公益电影免费放映等文化惠民工程。市博物馆新馆、莲花山公园免费对外开放。全民健身运动蓬勃开展，在全省第十四届运动会上取得22枚金牌的较好成绩。稳步推进保障性安居工程建设，筹资11亿元建设保障房6300套，基本解决拆迁安置历史遗留问题。发放住房公积金贷款6.63亿元，2100户家庭圆了住房梦。居民消费价格涨幅控制在2.2%。创新社会综合治理体制机制，深入排查化解社会矛盾，解决了一批影响社会稳定的问题。全面推进平安鄂州建设，社会治安防控体系不断加强，成为全省市州唯一连续3年命案全破城市。安全生产形势总体平稳，连续70个月未发生较大事故。改革食品药品监督管理体制，开展农村食品药品和城区散装食用油等专项整治，连续13年未发生食品药品安全事故。大力支持国防建设，广泛开展“双拥”活动，军政军民关系密切。社会保持和谐安定。

在推进经济社会平稳健康发展的同时，我们高度重视政府自身建设。一年来，政府系统认真开展党的群众路线教育实践活动，按照建设责任政府、法治政府、服务型政府、廉洁政府的要求，改进服务方式，转变工作作风。加强行政监察和审计监督，自觉接受人大、政协监督，认真办理人大建议和政协提案。严格执行国务院“约法三章”，“三公”经费下降40%。开展“六大专项整治”，集中解决了一批“四风”和关系群众切身利益的“最后一公里”问题，严肃查处了一批违法违纪案件。加强建章立制，规范权力运行，广大干部服务经济社会发展意识明显增强，依法行政水平明显提升。其它各方面的工作都取得了新的成绩。

　　各位代表，过去的一年，在十分困难的外部条件下，我们能够取得这些成绩实属来之不易。这是市委正确领导的结果，是人大代表、政协委员的有效监督、大力支持的结果，是全市人民凝心聚力、奋力拼搏的结果。在此，我代表市人民政府，向各位人大代表、政协委员，向各民主党派、无党派人士、工商联、人民团体和社会各界人士，向驻市人民解放军、武警官兵，向所有参与、支持、关心鄂州经济社会发展的同志们、朋友们，表示衷心的感谢和崇高的敬意！

　　在肯定成绩的同时，我们清醒认识到，全市经济社会发展还面临诸多困难和挑战。外部市场需求疲软，投资拉动作用发挥不够，资金、土地、用工等要素保障偏紧，部分企业经营困难，经济结构调整压力大，新的经济支撑点还不突出，财政增收难度较大，社会保障水平与民生需求还有一定差距。对这些问题，我们一定认真对待，积极采取有力措施，切实加以解决。

　　二、2015年工作安排

　　今年，是全面深化改革的关键之年，是全面推进法治鄂州建设的开局之年，也是全面完成“十二五”规划的收官之年。做好今年工作，意义重大，影响深远。当前，经济发展进入了速度换挡、结构优化、动力转换的新常态，新常态孕育着新机遇。我们要善于把握各种有利条件，主动适应和引领新常态。要充分认识到，我市仍然处于大有可为的“黄金发展期”，经济发展基础较好，有很大潜力，有较强的韧性；要看到，国家为应对经济下行压力实施定向调控、精准发力，采取一系列的政策组合拳有利于宏观经济的稳定发展；还要看到，随着国家长江经济带开放开发和全省“一元多层次”战略加快落实，我市的战略地位和比较优势将更加显现；特别是我们坚持“一改两化”不动摇，不断释放改革红利，强力推进新区开放开发，多极支撑、城乡协调发展格局已经形成，一大批重大项目纷纷落户，为我市科学发展、跨越式发展提供强大后劲和潜能。我们要以时不我待、奋发有为的精神状态，以勇于担当、创新务实的积极作为，全面完成“十二五”规划和年度目标任务，推动鄂州经济社会发展迈上新台阶。

　　今年政府工作的总体要求是：全面贯彻党的十八大、十八届三中、四中全会精神，坚持“稳中求进”工作总基调，坚持“竞进提质、升级增效”工作总要求，牢牢把握“市场决定取舍、绿色决定生死、民生决定目的”三维纲要，坚持“一改两化”不动摇，始终不渝抓改革，脚踏实地调结构，积极进取抓创新，竭尽全力保民生，不断创造鄂州速度、鄂州效率、鄂州标准、鄂州质量，努力为全省“建成支点、走在前列”作贡献、打头阵。

　　经济社会发展主要预期目标是：全市地区生产总值增长10%，规模以上工业增加值增长11%，全社会固定资产投资增长20%，社会消费品零售总额增长13.5%，地方公共财政预算收入增长12%，外贸出口增长10%，城镇常住居民人均可支配收入增长10%，农村常住居民人均纯收入增长11%，城镇登记失业率控制在4.5%以内，居民消费价格涨幅控制在3.5%以内，完成省定节能减排目标。

　　围绕以上目标，重点抓好以下七个方面工作。

　　（一）突出产业培育，夯实基础平台，全面提升新区开放开发水平。全面加快八大新区建设发展步伐，把新区作为抢抓国家长江经济带开放开发战略机遇的承接点、新型城镇化建设的支撑点，使新区成为开放型经济的新高地和转型发展的新引擎。一是明确发展定位，大力培育特色产业。立足各新区比较优势，明确产业主导方向，避免低水平重复和同质化竞争，走错位发展的路子。葛店开发区重点发展电力、高端装备制造、导航通讯、生物医药、电子商务等产业；鄂州开发区重点发展智能制造、新型建材、节能环保、通用航空、汽车服务等产业；鄂城新区和花湖开发区重点发展新能源、装备制造、文化体育等产业；三江港新区和红莲湖新区重点发展仓储物流、电子信息、特种汽车、休闲旅游等产业；梧桐湖新区和梁子湖生态文明示范区重点发展文化创意、生态农业、生态旅游等产业。使八大新区形成八仙过海、各显神通、竞相发展的生动局面。二是加快基础设施建设，着力提高承载能力。运用市场化方式推动新区基础设施建设和产业发展。按照“新区独立成篇”的思路，前瞻规划、科学布局、滚动推进。先行拉开主干路网骨架，优先建设生产配套设施，同步完善城市生活服务功能，确保八大新区全年完成投资300亿元以上，使新区生产配套设施与生活服务体系对接融合，提高项目和人口的承载力。创新拆迁、安置模式，实现新区人口就地市民化。三是坚持以产兴城，扎实推进产城融合。牢固树立产城互动理念，依靠大产业、大布局推动新区大发展。围绕新区产业定位，谋划一批重点产业项目，扎实推动项目落地，全力推进投资10亿元以上的鄂州电厂三期、南都新能源、南山临港新城、华威科、依讯北斗、彤诺电脑、新宇特种车、滨湖电子、迪卡侬、苏宁云商、上海易商、潮团冷链、港中旅等重大项目建设。促进主城区与八个新区之间的功能分工、协调互动，形成多点发力、共同发展格局，建设宜居宜业组群式大城市。四是强化招商引资，做大做强经济底盘。实施招商引资“一把手”工程，坚持大员上阵，专业队伍主动出击，上门招商。瞄准未来成长最快、潜力最大的领域和行业，抓好重点产业和重大项目的引进工作，每个新区要引进2个以上投资过10亿元的产业项目。注重招商引资质量，坚持科学发展一步到位，既注重引进实力强、规模大的支柱型大项目，又注重引进成长性好，产业关联配套能力强的科技型、创新型、集群型项目。严格投资强度、税收贡献程度，对污染重、能效低、安全生产保障难度大的项目坚决拒之门外，全年新洽谈引进亿元项目百个以上。

　　（二）突出先行先试，狠抓改革攻坚，不断释放发展红利。以厘清政府与市场、政府与社会的关系为突破口，以市场化改革为取向，以简政放权为先手棋，全面深化各领域改革，激活转型升级的内生动力。一是深化行政体制改革。推进简政放权，深化行政审批制度改革，全面清理规范非行政许可审批事项，实行政府部门职责清单、审批清单、投资负面清单、收费清单制度。加强新区行政审批管理服务承接能力建设。进一步放开市场准入条件，推行工商、公安、质监、国（地）税联合办证和“多号合一”试点，激发全民创业活力。二是深化投融资体制改革。创新PPP合作模式，探索“一区多园”建设模式，完善沿江滨湖新区开发机制。建立投资项目在线审批监管平台及网上并联审批制度，推进投资便利化。加快发展多层次资本市场，鼓励企业在创业板、“新三板”、武汉股权托管交易中心挂牌上市，面向资本市场直接融资。深化公共资源交易改革和行政事业性国有资产管理运营改革。加快推进新一轮国企改革。三是深化财政体制改革。推进预算管理改革，进一步健全预算体系和预算标准体系，严格执行收支两条线和国库集中收付，进一步理顺市、区、乡镇财政管理体制，提高资金使用效率。四是深化农村产权制度改革。全面完成土地确权颁证工作，推动农村土地所有权、承包权、经营权三权分置。完善农村产权交易市场体系，把农村土地经营权等“五权”流转交易全部纳入农村产权交易平台，建立市、区、乡、村四级联网的交易信息系统。五是创新生态文明体制机制。建立自然资源资产管理制度，明确自然资源资产监管职责权益，完善自然生态空间统一确权登记制度。探索建立资源有偿使用和生态补偿机制，开展碳排放权、排污权交易试点和抵押融资。

　　（三）突出高端导向，推进转型升级，加快构建现代产业体系。深刻把握新常态下产业形态更高级、分工更复杂、结构更合理的演化趋势，聚焦新技术、新产品、新业态、新模式，以做大增量、调优存量来引领新常态，推动转型发展。一是促进传统产业改造升级。研究制定符合产业发展方向的政策措施，充分聚合政府引导、市场倒逼、企业主体、科技引领作用，引导传统产业改造升级。推动信息化与工业化融合，提升传统工业信息化、智能化水平。用先进适用技术改造提升传统产业，实行清洁生产，实现产品结构调整、质量提高、效益提升。引导企业资产重组、兼并联合，鼓励龙头企业做大做强，中小企业投大靠强。深入实施质量强市战略，打造一批在国内外有影响的拳头产品、著名商标、名牌企业。发挥市中小企业担保公司作用，切实缓解中小企业融资难题，支持中小微企业发展。二是推动高新产业规模化。深入实施创新驱动战略，加强创新服务体系建设，加快建设科技加速器、孵化器、创新平台和创业园区，建立鼓励大众创业、万众创新的体制机制，在葛店开发区打造科技人员、高校学生创业创新的“梦工厂”。运用高新技术产业基金的引导和杠杆作用，引进各类风投基金、股权基金，扶持吸引科研成果在我市转化落户，实现产业化和集群化。建立产学研合作机制，制定配套措施，鼓励企业技术创新，推动形成一批科技型企业群体。全年新增规模以上工业企业50家以上。三是积极培育转型发展新支柱。着眼“十三五”，超前谋划，提早布局，突破性引进、培育、发展智能制造、机器人、通用航空及服务、电脑制造及信息服务等高端产业项目。实行大员上阵，一个项目一个专班，用钉钉子精神，全力推进，努力为鄂州经济转型和未来发展提供新的产业支撑。四是加速发展现代服务业。实施第三产业倍增工程。加快葛店开发区“国家电子商务示范基地”建设，构建大仓储、大物流、大智慧支撑的大电商基地，努力争取跨境电子商务交易试点获批。引进1-2家金融机构，创新金融品种，提高金融对实体经济服务能力。着力扩大对外贸易，鼓励企业扩大出口规模，支持企业开展对外工程承包和劳务合作，拓展发展空间。充分发挥三江港铁路货运港航枢纽的独特优势，积极引进有实力、有运作能力的物流企业，大力发展临港经济，把三江港和葛店港打造成为长江中游对外开放的窗口和开放型经济的高地。推进梁子湖“一心两翼”生态旅游和红莲湖“印记武汉、欢乐红莲”等项目建设，打造鄂州旅游升级版。

（四）突出生态农业，强化“三农”基础，深入推进城乡发展一体化。巩固农业基础地位，转变农业发展方式，探索出一条产出高效、产品安全、资源节约、环境友好的生态农业发展道路。一是推动生态农业创新提质增效。加快“一区三线四园”建设。发挥峒山全国现代生态农业创新基地、台湾生态农业创业园、生态渔业示范区等生态农业基地的示范引领效应，推进全市生态农业标准化示范区和梁子湖有机产品认证示范区建设，新建生态农业基地50个。坚持打生态有机牌，走特色优质路，实施品牌兴农战略，加快“三品一标”农产品认定认证。二是推进适度规模经营。鼓励发展农村合作经济，改变传统农户经营规模小、分散粗放状况，发展集约规模经营。大力培育专业大户、家庭农场、农民合作社、农村专业技术协会等新型农业主体，新增农民专业合作社50家。尊重群众意愿，保障农户利益，加强耕地保护，引导土地向新型农业主体适度集中，力争全市土地流转率达到38%。建立农业社会化服务体系，培养新型职业农民。加强排灌系统、路网电网、国土整治、生态大棚、信息化等设施建设，加强农业服务“新网工程”建设，打通农业生产服务“最后一公里”。三是大力发展农产品加工。发展壮大农业加工企业，依托龙头企业推进农业产业化，提升农产品加工转化能力。扶持长链条、深加工、带动强的重点企业提档扩规，确保全市农产品加工业产值增长15%以上。用好生态农业发展资金，扶持和推动生态农业和农业产业化发展。建立完善农产品质量认证、在线查询、网络化销售、专业化运营的营销体系，全市60%农业龙头企业实现网络销售。四是加快城乡一体化进程。进一步完善体制机制，做实城乡基本公共服务体系，提升服务水平。进一步优化乡村治理结构，完善农村社会管理，按照生产、生活、生态和谐的理念，建设美丽乡村。加快推进首批30个村示范点创建工作，实施“田园宜居、乡村文化、通畅路网、环境治理、乡村绿化”五大工程，彰显自然、生态、环境、地域特色。深入实施“清洁乡村”工程，加快农村污水处理设施建设，连线成片推进村庄环境综合整治。深入开展“三万”活动和城乡结对帮扶活动。

　　（五）突出完善功能，创新建管机制，提升城市内在品质。把握城市发展规律，将城市规划建设与管理的重心转移到完善设施功能、优化人居环境、提升城市品质上来，让广大市民感受现代城市的亲和力、宜居度和认同感。一是打通节点，补齐短板。按照全域一体，集中力量推进基础设施互联互通，加快吴楚大道西段、东段、葛山大道改扩建、主城区客运枢纽站等重点工程建设，开展凤凰大桥、鄂咸高速建设。打通主城区与各新区之间、各个新区之间、新区内部之间的交通节点，推进城乡客运一体化，实现“村村通客车”。二是完善功能，提升品质。满足城市多层次生产生活、精神文化需求，推进三国吴都风光带五期、天龙山公园、吴都中央生态廊道等园林绿化工程建设。开展市民中心、文化中心、体育中心和群艺馆、科技馆等项目前期工作和建设。完善城市交通、停车场、绿化、集贸市场等公共设施建设。加快火车站商圈建设。推进鄂州职业大学校中村、朱家垴、水泥厂等棚户区改造和旧城区改造。完善和提高城乡供水一体化水平，实施雨台山水厂、太和水厂改扩建工程。认真做好“多规合一”试点工作。三是突出重点，强化管理。推进“五城同创”工作常态化、全域化。主城区各项治理工作从主次干道、商业大街向背街小巷延伸；从中心区域向城中村、城乡结合部延伸；从有人管理区域向无人管理的区域延伸。各区和中心镇集中力量建设停车场、菜市场、小商品市场等疏导场所。推进智慧城市、智慧城管建设，开展“示范街”创建活动。实施背街小巷、主城区旱厕改造，加快洋澜湖综合治理及老城区雨污分流工程建设。巩固物业管理全覆盖成果，提升管理服务水平。持续开展市容市貌、环境卫生、交通秩序等城市环境综合整治。继续抓好“禁鞭”工作。深入开展“百万市民素质提升工程”，树立良好社会风尚。

　　（六）突出绿色发展，强化生态优势，建设生态文明先行区。牢牢把握“绿色决定生死”的深刻内涵，把尊重自然、顺应自然、保护自然的理念融入经济社会发展全过程，把生态优势转化为发展竞争优势。一是大力推进环境整治。深入实施蓝天、碧水、净土工程。继续采取工程减排和管理控排措施，加强重点工业企业污染治理和实时在线监管，实施鄂钢、球团厂、华新水泥粉尘治理工程。扩大强制性清洁生产企业范围，淘汰落后工艺设备，落实企业主体责任，确保控排设施与生产同步、匹配运行。依法关停一批污染重的小粉磨、小窑炉、小铸造、小采石、小矿山等小企业。综合治理工业废气、机动车尾气和工地扬尘三大污染源，减少灰霾天气。建立大气污染和极端天气预警制度。强化农业点源面源污染防治，全市规模养殖场要建立治污设施。二是打造生态文明优势。以创建梁子湖生态文明示范区、全国水生态文明示范市为契机，加快实施梁子湖流域综合治理防洪一期、洋澜湖生态补水等工程，全市水质监测中心建成投入使用。坚决制止围湖养殖，用3年时间完成全市主要湖泊退池还湖、破堤还湖工作。推进城乡一体垃圾、污水全收集全处理体系建设。开展绿满荆楚行动，积极创建林业生态示范区，推进汉鄂高速、吴楚大道沿线绿化林带建设，完成4.5万亩造林任务，实现2年“绿满鄂州”的奋斗目标，突显“山川秀美、百湖之市”的生态鄂州优势。三是探索绿色发展新路径。严格执行项目环境评价、节能评估和“三同时”制度，引入绿色准入机制。依托项目建设，用足鼓励政策，加强光伏太阳能、生物质能规模化开发利用。提高LNG、管道燃气在一次能源消费中的比重，实施主城区、新区锅炉“煤改气”工程，支持内湖船舶、道路运输车辆“油改气”。推进资源综合利用，大力发展循环经济。加强工矿区、砖厂、小选厂废弃土地修复和再利用。大力推广建筑、交通运输、公共机构节能新技术、新模式，倡导低碳生活、绿色消费新观念。

　　（七）突出民生目的，坚持底线思维，使发展成果更多惠及人民群众。把改善民生作为政府第一责任，顺应人民群众的新需求、新期待，守住底线、突出重点、完善制度，全力办好民生实事，不断提升全市人民幸福感。一是推进创业促就业。坚持扩大就业和优化就业结构并重，提升下岗失业人员、新生劳动力和农村富余劳动力的创业就业能力，鼓励支持大学生自主创业，确保全年城镇新增就业1.8万人。二是提高城乡社会保障水平。继续提高退休职工养老金标准，提高城乡居民基本养老保险基础养老金标准。做实“五类”特困群众兜底保障工作，确保困难群众基本生活。完善社会救助体系，提高救助覆盖面和救助水平。推进全民社保登记工作。完成49个城乡社区居家养老和互助养老服务项目建设。建设公租房728套，为低收入住房困难家庭发放廉租房补贴。三是办好人民满意教育。深化教育领域综合改革，实施义务教育薄弱学校改造、教育信息化、中等职业教育、农村学前教育推进工程，完成葛店高中、市学生实践基地一期建设。支持鄂州职业大学提升办学质量和层次。推进华师大鄂州校区、葛店职教园建设。加强全市社会教育培训机构管理。四是提升医疗卫生服务能力。建立城乡一体的远程诊疗系统，推进基本医疗服务下沉。加快市中心医院综合楼、市二医院新院、市三医院改扩建工程建设。推进公立医院改革。做好重大传染病防控，完善各项应急预案。大力开展出生人口性别比综合治理，加大“两非”查处，稳定低生育水平。五是大力发展文化体育事业。加快推进现代公共文化服务体系建设，鼓励、吸纳社会资本参与公共文化体育设施建设。推进吴王城遗址保护工作。提高文化场馆服务水平。继续开展全民健身运动和周周乐、周周演、送戏下乡等文化活动，推进社区（村）公益电影固定放映点建设和管理。六是加强社会治理。深化法治鄂州、平安鄂州建设，加强社会治安综合治理，健全城乡一体网格化管理服务体系，完善立体化治安防控体系，创新社会矛盾纠纷化解机制，推进阳光信访信息系统建设，切实维护人民群众的合法权益。加强安全生产，落实安全生产责任，加大对重点行业、企业安全隐患排查整治力度。加强食品药品监管，严厉打击制售假冒伪劣食品行为，加强大众饮食摊点卫生整治，推进放心粮油市场体系建设。打击和处置非法集资，确保社会和谐稳定。认真做好外事侨务和民族宗教工作。进一步落实国防动员法、兵役法等法规，实现国防建设和经济建设同步发展。

　　三、加强政府自身建设

　　人民群众的期盼就是政府努力的方向。新的一年，我们将深入巩固拓展群众路线教育实践活动成果，以建设责任政府、法治政府、服务型政府和廉洁政府为目标，坚持依法行政，全心全意为人民服务，以实实在在的业绩，赢得人民群众的信任。

　　（一）坚持执政为民。把人民群众满意作为衡量政府工作的唯一标准。始终将群众利益摆在首位，牢固树立正确的政绩观，多做打基础、管长远、利当前、惠民生的工作。深入基层、深入群众，用心体察民情，诚心诚意为群众办实事、解难事、做好事。按照“清廉为官、事业有为”要求，大力提升“精神区位”，进一步转变作风，勇于负责，敢于担当，努力增进全市人民福祉。加强制度建设，完善绩效考评，整治懒政、怠政、为官不为等不良风气，使各级政府和部门的精力用到谋发展、抓落实上。实行“一线工作法”，确保重大决策、重大项目落地有力，重点工作高效推进。整合亲民热线、市长专线、电视问政、微信微博等政民互动平台，保障群众知情权、参与权。进一步推进公共服务下沉，让群众少跑腿、少受累。

　　（二）坚持依法行政。把依法行政贯穿于政府工作的全过程，建设职能科学、权责法定、执法严明、公正公开、守法诚信的法治政府。规范行政行为，严格按法定权限和程序履职，破除把依法行政与提高办事效率对立起来的观念，进一步提高行政效能。严格执行人大及其常委会的决议决定。全面清理规范行政自由裁量权，建立健全行政裁量权基准制度。严格执行重大决策和规范性文件合法性审查和廉洁性评估。推进行政执法体制改革，全面落实行政执法责任制，着力解决多头执法、多层执法和权责交叉的问题，促进公正、严格、文明执法。实行法律顾问制度，建立覆盖城乡的基层法律顾问服务体系。加强行政权力运行监督，完善政府监管清单，明确政府部门监管职责，提高政府治理能力和水平。抓好“六五”普法，提升全民学法尊法守法用法的自觉性。

　　（三）坚持科学理政。进一步转变政府职能，充分发挥市场在资源配置中的决定性作用，把该放的权放到位，把该管的事管住管好。健全科学决策、民主决策机制，严格执行专家论证、风险评估、公众参与、集体讨论决定的程序。精心编制好经济社会发展“十三五”规划。认真办理人大建议和政协提案，广泛听取民主党派、无党派人士、工商联、人民团体的意见建议。深入推进阳光政务，及时公开财政预算、公共资源配置、重大投资项目、社会公益事业等政府信息，提高政府工作透明度。引入竞争机制，规范政府购买服务行为。加快推进信用体系建设，建立诚信公平的市场经济秩序，为经济社会发展创造良好环境。严格政府部门行政问责、行政过错责任追究制度。加强对各项工作落实的监督，确保落实到位。

　　（四）坚持廉洁从政。全面落实党风廉政建设责任制，严格履行主体责任，坚持“一岗双责”和“力度统一论”，进一步加强政府系统党风廉政建设和惩防体系建设，保持惩治腐败高压态势，对腐败“零容忍”。强化政府重大投资项目、重大财政资金支出的审计和监管，严格农村“三资”管理，规范乡镇财政管理，严肃查处违法违纪案件。自觉接受人大法律监督、政协民主监督、社会公众和新闻舆论监督。厉行勤俭节约，降低行政运行成本，严格控制“三公”经费，推进公务用车制度改革，把有限的资源和财力用在推动发展和改善民生上，让政府过紧日子，让百姓过好日子。坚决反对“四风”，着力解决群众反映突出的政风行风问题，树立为民务实清廉的良好形象。

　　各位代表，新的一年已经开始，改革发展的任务光荣而艰巨。让我们高举中国特色社会主义伟大旗帜，认真贯彻落实党的十八大和十八届三中、四中全会精神，在省委、省政府和市委的坚强领导下，紧紧依靠和团结全市人民，解放思想，改革创新，凝心聚力，克难攻坚，埋头苦干，为全面建成小康社会而努力奋斗！
